# Maternal obesity alters methylation level of cytosine in CpG island for epigenetic inheritance in fetal umbilical cord blood

**DOI:** 10.1186/s40246-022-00410-2

**Published:** 2022-08-31

**Authors:** Zhuoyao Ma, Yingjin Wang, Yanmei Quan, Zhijie Wang, Yue Liu, Zhide Ding

**Affiliations:** 1 Department of Histology, Embryology, Genetics and Developmental Biology, Shanghai Key Laboratory for Reproductive Medicine, Shanghai Jiao Tong University School of Medicine, No.280, Chongqing Road (South), Shanghai, 200025 China; 2 Department of Obstetrics and Gynecology, Shanghai Eighth People’s Hospital, Shanghai, 200235 China

**Keywords:** Maternal obesity, Umbilical cord blood, Epigenetics, Differentially methylated region (DMR), DNA Methylation, Pathways

## Abstract

**Background:**

Over the past few decades, global maternal obesity prevalence has rapidly increased. This condition may induce long-lasting pathophysiological effects on either fetal or infant health that could be attributable to unknown unique changes in the umbilical blood composition.

**Methods:**

A total of 34 overweight/obese and 32 normal-weight pregnant women were recruited. Fifteen umbilical blood samples including 8 overweight/obese subjects and 7 normal weight women were sequenced using Targeted Bisulfite Sequencing technology to detect the average methylation level of cytosine and identify the differentially methylated region (DMR). GO and KEGG analyses were then employed to perform pathway enrichment analysis of DMR-related genes and promoters. Moreover, the mRNA levels of methylation-related genes histone deacetylases (HDACs) and DNA methyltransferases (DNMTs) were characterized in the samples obtained from these two groups.

**Results:**

Average methylated cytosine levels in both the CpG islands (CGI) and promoter significantly decreased in overweight/obese groups. A total of 1669 DMRs exhibited differences in their DNA methylation status between the overweight/obese and control groups. GO and KEGG analyses revealed that DMR-related genes and promoters were enriched in the metabolism, cancer and cardiomyopathy signaling pathways. Furthermore, the HDACs and DNMTs mRNA levels trended to decline in overweight/obese groups.

**Conclusions:**

Decreased methylated cytosine levels in overweight/obese women induce the gene expression activity at a higher level than in the control group. DMRs between these two groups in the fetal blood may contribute to the changes in gene transcription that underlie the increased risk of metabolic disorders, cancers and cardiomyopathy in their offspring.

**Supplementary Information:**

The online version contains supplementary material available at 10.1186/s40246-022-00410-2.

## Background

Recently, obesity is gaining more worldwide attention because it is becoming apparent that it contributes to the increased incidence of metabolic diseases, such as diabetes, heart disease, fatty liver, arteriosclerosis, and varied tumors [1, 2]. A large number of animal experiments and clinical studies have shown that the offspring of obese mothers also have an increased incidence of health-related issues. Both pre-gestational obesity and excessive weight gain during pregnancy are critical risk factors for developing various adverse fetal outcomes [3]. For instance, maternal obesity may increase the risk of postnatal obesity and cardiovascular diseases in adolescent children [4–6]. Moreover, a recent study showed that a multi-generational maternal exposure to a high-fat diet can cause increased incidence of hepatocellular carcinoma (HCC) in offspring [7]. In our previous study, we found that neonatal weight was positively correlated with pre-pregnancy BMI in the pregnant women [8]. However, the underlying biological mechanisms remain unclear.

Epigenetic studies deal only with characterizing the roles of modifiers of gene expression levels in inducing phenotypic changes. Such control is mediated through modulation of DNA methylation status, histone alterations, genomic imprinting, chromosomal rearrangement and changes in noncoding RNA side groups to mediate intergenerational phenotypic transmission [9, 10]. Among them, DNA methylation is the earliest and most widely studied modulation identified in different clinical disciplines or basic science topics. DNA methylation refers to the process in which organisms use s-adenosylmethionine as a methyl donor to transfer through DNMT catalysis methyl groups to specific nucleotide sites. Such control is modulated by changes in the DNA methylation status at a gene or promoter. They can suppress or activate the readout of a gene controlling the expression of a phenotype [11].

DNA methylation status changes contribute to regulating many biological processes by regulating the transcription of genes involved in controlling cell proliferation, metabolism, apoptosis, invasion and migration. They underlie responses that account for various diseases [12]. DNA methylation is an inhibitory epigenetic marker associated with stable gene silencing. During the early differentiation and developmental stages of the mammalian embryo, the genome undergoes a process of demethylation and then re-methylation. For example, maintenance of the mature sperm and oocyte phenotype depend on sustaining a high DNA methylation status before fertilization. After fertilization, the DNA methylation status declines reaching its lowest level at the 8-cell stage and then re-methylation rapidly occurs, reaching a somatic level at the blastocyst stage [13–15].

Fetal development is modulated by changes in epigenetic phenomena that include changes in stage dependent modification of the expression profile of DNA methylation status, histone modification, and alteration of noncoding miRNA patterns [16–18]. The maternal obesity phenotype impacts on fetal and infant development through modulating epigenetic changes. On the other hand, the umbilical cord allows for establishment of a unique exchange pathway between the maternal and fetal circulations. To probe the epigenetic changes in umbilical cord blood of newborn from the obese pregnant women, especially the changes in DNA methylation in blood cells can greatly help us to understand how the maternal obese environment affects fetal gene expression patterns that are established during development. Accordingly, we sequenced different fetal umbilical cord blood samples from normal weight and overweight/obese women and describe here how changes in the DNA methylation status and the pathway enrichment of DMR-related genes and promoters.

## Methods

### Enrollment of subject

Chinese pregnant women were recruited for their first prenatal checkup at the Shanghai Eighth People's Hospital before the end of the first trimester (< 12 weeks). These samples were from non-smoking pregnant women without gestational diabetes and any other complications: a total of 66 overweight/obese (BMI ≥ 25 kg/m^2^, *n* = 34) and normal weight (18.5 kg/m^2^ < BMI < 25 kg/m^2^, *n* = 32) pregnant women were selected. The ages of the overweight/obese group and the control group were 29.47 ± 0.55 years and 28.88 ± 0.65 years, respectively. The study was approved by the Institutional Review Committee of Shanghai Eighth People's Hospital (No. 2019002), and all subjects gave informed consent before participating.

### The genomic DNA extraction

The genomic DNA was isolated using the PAXgene Blood DNA kit (Qiagen, Germany) and the extraction process followed the procedure described in the PAXgene blood RNA kit handbook. Briefly, all blood in a PAXgene Blood DNA tube was poured into a tube containing 25 ml BG1 buffer. After mixing, the tube was centrifuged at 2500 xg for 5 min and the supernatant was discarded. Then, 5 ml BG2 buffer was added into the tube, and the above steps were repeated. Next, 5 ml BG3 buffer /PreAnalytiX Protease was added and the tube was incubated at 65 °C for 10 min, and then vortexed at high speed for 5 s. Subsequently, 5 ml isopropanol was added to precipitate DNA and the tube was centrifuged at 2,500 xg for 3 min and washed with ethanol. Then, ethanol was removed and the DNA pellet was dried for 5 min. Finally, 1 ml BG4 buffer was added and incubated at 65 °C for 1 h to dissolve the DNA.

### Whole genome and site-specific DNA methylation analyses of cord blood

The Beijing Genomics Institute (BGI, Beijing, China) performed bisulfite sequencing in the target genomic area of the umbilical cord blood samples (control group = 7, including 3 male and 4 female newborns; overweight/obese group = 8, including 4 male and 4 female newborns). After filtering low-quality reads, N read and adapter sequence, an average of 10.000 Gb clean bases were generated. Then, MAPping (BSMAP) (http://code.google.com/p/bsmap/) bisulfite sequence analysis was performed to map the clean reads to the reference sequence. Strict quality control was carried out on several QC terms for each sample to confirm the identity of the sequencing data. The methylation level of each gene was analyzed when the promoters were covered by at least 5 cytosine phosphate-guanine (CpG) sites. Meanwhile, the methylation status of the genes was recorded on each chromosome. This procedure included evaluating the mean methylation level of covered cytosines throughout the gene including the promoter regions.

### Bioinformatics analysis of differentially methylated regions

The reads which covered cytosine determined the methylation level [19]. They were also equal to the mC/C ratio at each reference cytosine [20]. Methylome comparisons of the samples of the control group and overweight/obese group identified putative DMRs. This was done by using windows that contained at least 5 CpG sites with a twofold change in their methylation level and Fisher test *p* value < 0.05. Both samples were included if the DMR discovery showed that they were not hypomethylated. Two nearby DMRs were considered interdependent and joined into one continuous DMR if the genomic region from the start of an upstream DMR to the end of a downstream DMR also had a twofold difference between their methylation level in samples of the control group and the overweight/obese group with a *p* value < 0.05. Otherwise, the two DMRs were viewed as independent. After iteratively merging interdependent DMRs, the final dataset of DMRs contained only those that were independent from each other [21, 22]. The methylation level of each gene was analyzed when at least 5 CpGs covered the promoters. Then, we assigned the methylation information to genes on each chromosome, including the mean methylation level of covered cytosines for the promoter, and mean methylation level of covered cytosines for the gene. Finally, the degree of difference was calculated between the mCpGs in two groups. CIRCOS was performed to compare the methylation level of DMR in different groups. The formula used to make this comparison is: degree of difference = log_2_Rm1/ log_2_Rm2. Rm1、Rm2 represents the methylation level of methyl-cytosine for group1 (normal-weight group) and group2 (overweight/obese group), respectively. 0.001 will replace Rm1(or Rm2) while it equals 0 [23].

Gene Ontology is based on GO TermFinder (http://www.yeastgenome.org/help/analyze/go-term-finder). This procedure compares the DMR-related genes and promoters with the GO term database (http://www.geneontology.org/). Subsequently, the number of genes are calculated in each term, and then subjected to the hypergeometric test to identify the GO terms that are significantly enriched in DMR-related genes and promoters by comparing them with the background of the entire genome. KEGG is the main public database that was applied to resolve the pathway identity (http://www.genome.jp/kegg/). In other words, significant pathway enrichment analysis usually uses KEGG as a tool to delineate the pathway that is significantly enriched in DMR-related genes relative to the entire genome background. The calculation method of KEGG is the same as GO analysis.

### RNA isolation from cord blood

RNA was purified from umbilical cord blood collected in the PAXgene blood RMA tube (Qiagen, Germany). The purification process followed the procedure described in the PAXgene blood RNA kit handbook. Briefly, the Blood RNA tube was equilibrated at room temperature for at least 2 h to ensure complete lysis of blood cells. Then, the RNA containing tube was centrifuged at 3000 to 5000 xg for 10 min at 15–25 °C to extract RNA. Next, RNA purification based on silica membrane was performed according to the manufacturer's protocol. The RNA concentration and purification were detected with the nanodrop 1000 spectrophotometer (Thermo, USA) and the purified RNA samples were stored at − 70℃ immediately for later experiments.

### Quantitative real-time PCR (RT-qPCR) analysis

Complement DNA (cDNA) was synthesized following the PrimeScript RT reagent kit (Takara, Japan) and the total cDNA was amplified with the TB Green Premix Ex Taq (Takara, Japan) with the 7500 real-time PCR system (ABI, USA). Sequences of primers used for RT-qPCR analysis are listed in Additional file 1: Table 1.

### Statistical analysis

Normally distributed continuous data are expressed as mean ± SE and are compared using Student's *t* test. The chi-square test was used to compare categorical data. When *P* < 0.05, the difference was considered to be statistically different.

## Results

### Average methylation level analysis

**Fig. 1:**
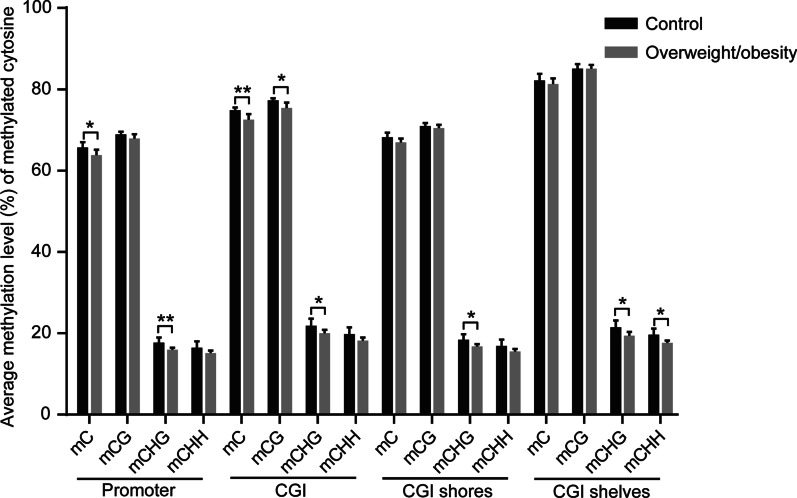
Analysis of average methylation level of cytosine. The average methylation status of cytosine in the promoter regions on cytosine islands CGI, CGI shores and CGI shelves. The DNA samples obtained from the umbilical cord blood in the two different groups were sequenced by Targeted Bisulfite Sequencing technology

After giving birth, the umbilical cord blood of newborns from 15 cases including 7 normal weight pregnant women and 8 overweight/obese pregnant women were randomly selected for DNA methylation level of cytosine sequencing. Each subject from a total of 15 cases underwent an individual independent analysis of DNA methylation status. Some studies showed that not only methylation of cytosine phosphate-guanine (CpG, also known as CG), but the non-CpG contexts (mCHG and mCHH, where H = A, C or T) also appears in the human embryo [24]. This study describes the proportions of mCpG, mCHG and mCHH formation in the total methylated cytosine content of newborn cord blood. The proportion of non-CpG methylation sites is less than 20% (Additional file 2: Table 2). Sample number three had large deviations, which precluded their inclusion in the control group during the all following analyses. We compared the average methylation level of cytosine (C), CpG, CHG and CHH in promoter, CGI, CGI shores and CGI shelves, respectively. The results documented no significant changes of mC in CGI shores and CGI shelves between the normal weight control group and overweight/obese group, whereas the average methylation level in the promoter and CGI in the overweight/obese group was significantly lower than that in the normal-weight control (Fig. 1). Thus, lower methylation status of cytosine in promoter and CGI in the umbilical blood obtained from the obese group suggests that the gene expression level in the blood cells was greater than in the control group [8]. This difference could contribute to the dysfunctional biological processes underlying the pathophysiology of various diseases in the offspring of overweight/obese pregnant women.

### Differentially methylated regions

**Fig. 2:**
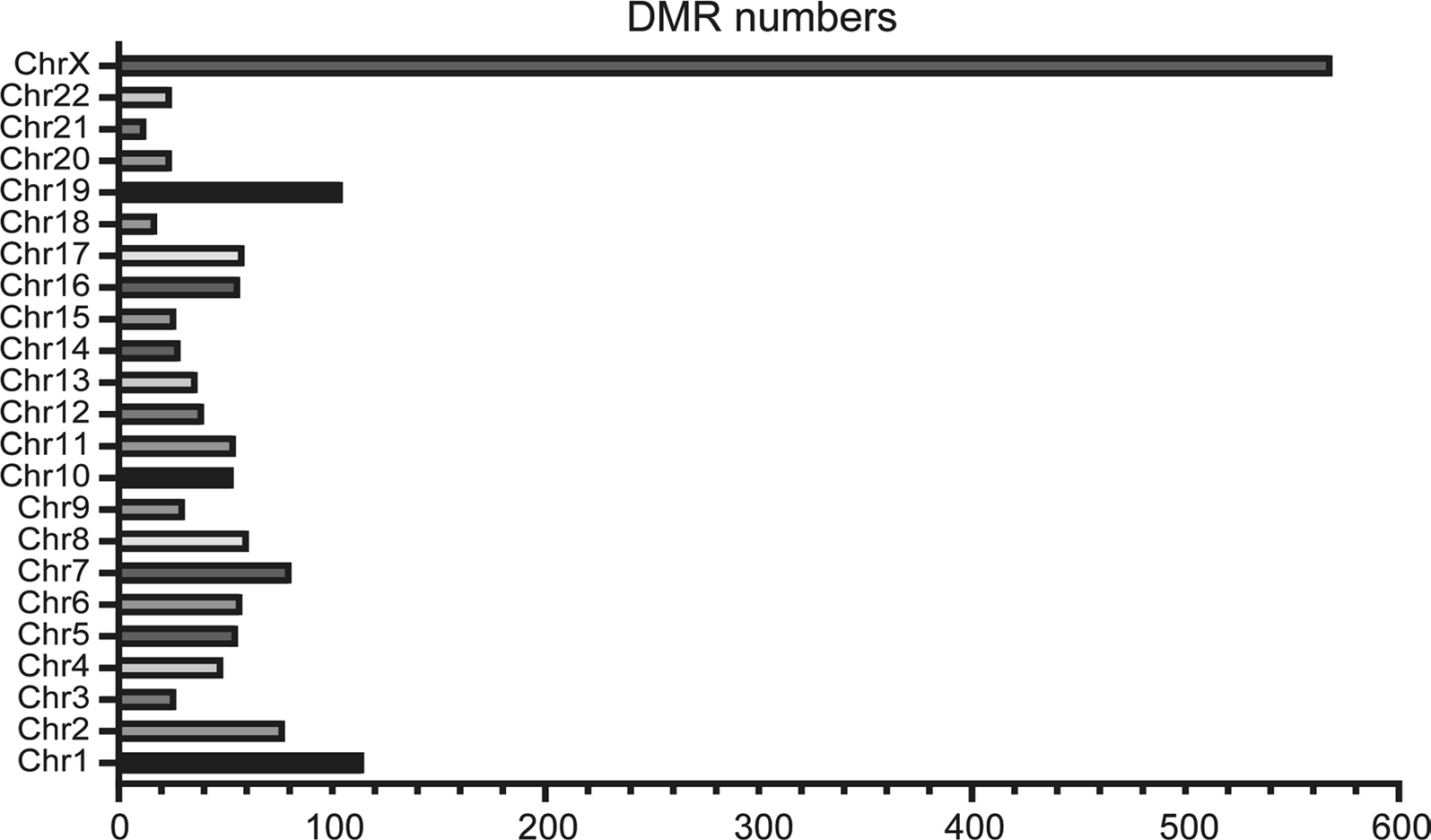
Differentially methylated regions. The number of differentially methylated regions on different chromosomes

Based on above results, we focused on methylation of cytosine in CpG island for further analysis. Putative DMR methylome comparisons were identified of the samples from the control group and the overweight/obese group using windows that contained at least 5 CpG sites with a twofold change in their methylation level and Fisher test *p* value < 0.05. A total of 1669 DMRs exhibited differences in their DNA methylation status between the overweight/obese group and normal weight control group (Additional file 3: Table 3). If the 569 DMRs were excluded that are located in the X chromosome, the remaining difference of 1100 was appreciable (Fig. 2). Some DMR-related genes and promoters differences that presented between the normal weight control group and overweight/obese group were listed in Additional file 4: Table 4 and Additional file 5: Table 5.

Illustrating the results in column chart shows that differences in methylations were more evident on chromosomes 1, 2, 7, 19. Moreover, nearly 6.8% and 6.2% of the DMRs at CpG sites were located on chromosomes 1 and 19 (Fig. 2) in the overweight/obese group.

### Pathway analysis on differentially methylated regions

**Fig. 3:**
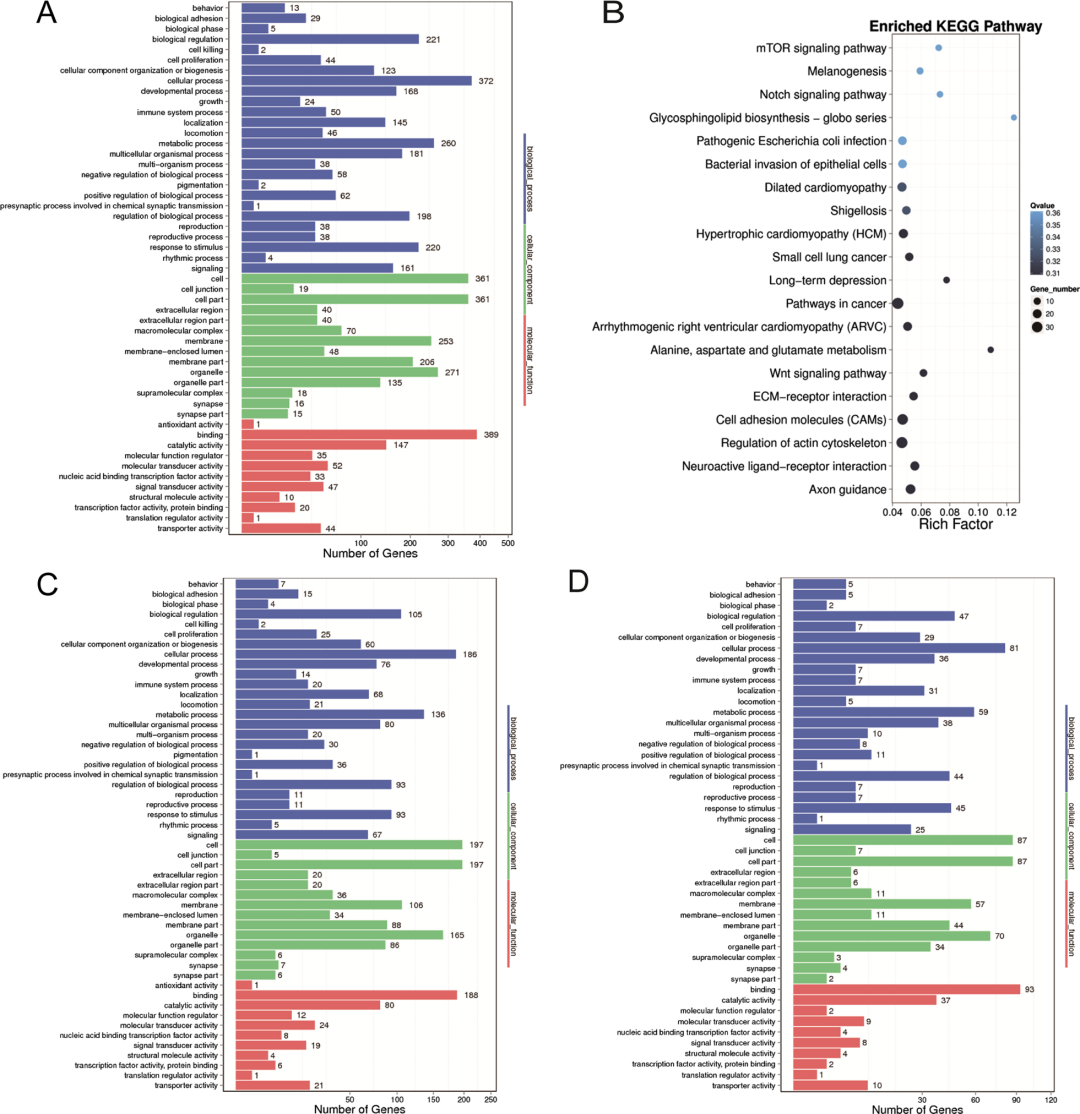
Pathway Analysis on DMR-related genes and promoters. GO and KEGG enrichment of differentially methylated regions. The molecular functions, cellular components and biological processes were investigated by GO analysis of DMR-related genes **A** and promoters **C** between the control and overweight/obese groups. **B** Scatter plot of KEGG pathway enrichment for the DMR-related genes. **D** GO analysis of DMR-related genes on all of the chromosomes except for those on the X chromosome

DMR-related genes and promoters results were then enriched for use in the GO and KEGG pathways analyses. GO analysis includes characterizing their possible molecular function, cellular component and biological process involvement, which are shown in Figs. 3A and 3C. Herein, we focused on the impact of maternal obesity on the biological process of offspring. The analysis of the DMR-related genes and promoters that underlie this process revealed many genes are involved in controlling cellular, metabolic processes and biological regulation. Notably, there were 260 genes and 136 promoters associated with the obtained DMRs results, which are relevant to a metabolic process, implying that there is a significant correlation between maternal obesity and an acquired metabolic disorder in the offspring.

Moreover, the DMR-related genes overlapped with 20 KEGG pathways, which were potentially associated with diseases (Fig. 3B). The pathway analyses of DMR-related genes indicated their involvement in cancers (small cell lung cancer), cardiomyopathy (hypertrophic cardiomyopathy, arrhythmogenic right ventricular cardiomyopathy and dilated cardiomyopathy) and nervous system-related signaling pathways (neuroactive ligand-receptor interaction and axon guidance). As many DMRs were identified on the X chromosome, GO analysis was also performed of DMR-related genes on all of the chromosomes except for those on the X chromosome (Fig. 3D). However, these results were similar to those obtained on all the chromosomes (shown in Fig. 3A).

In conclusion, maternal obesity may disrupt the glucose and lipid metabolic processes and even impose a severe disease risk for developing diseases, such as cardiomyopathy and cancer in the offspring.

### The expressions of HDACs and DNMTs showed decreased-trends in overweight/obese group

**Fig. 4:**
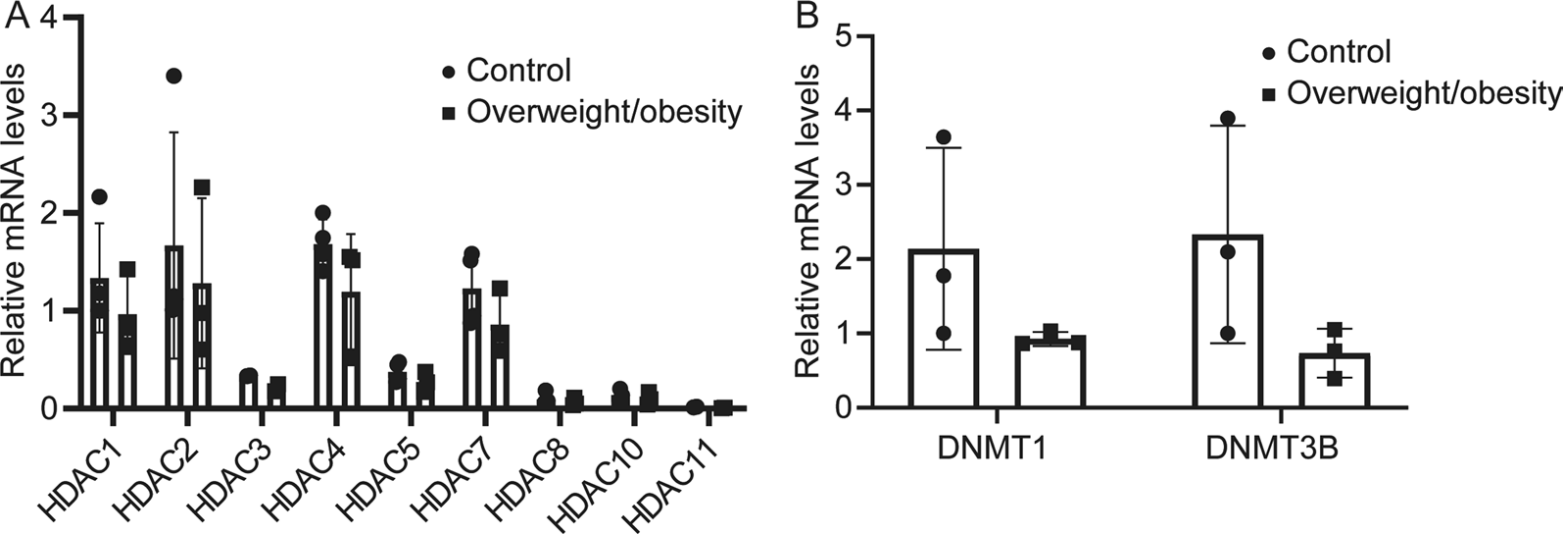
The mRNA level of HDACs and DNMTs. HDACs **A** and DNMTs **B** expression levels in fetal umbilical cord between the normal weight control group and overweight/obese group. RT-PCR results indicate that both DNMTs and HDACs had downward trends in overweight/obese group

Finally, we detected the mRNA levels of HDACs (Fig. 4A, Additional file 6: Table 6), DNMT1 and DNMT3b (Fig. 4B, Additional file 6: Table 6). Although the individual differences between the obese and the control groups were not significant, both HDACs, DNMT1and DNMT3b underwent obvious downward trends in the overweight/obese group. Generally, high expression levels of HDACs, DNMT1 and DNMT3b can inhibit gene transcription [25–29]. Moreover, these results were consistent with our finding that methylation levels of cytosine in the promoter and the CGI were significantly lower in overweight/obese group compared to those in their normal weight counterpart. This difference is supportive of the notion that in the obese group gene expression activity is at a higher level than in the control group.

## Discussion

In recent years, it is becoming increasingly apparent that maternal obesity can impair normal fetal cell development. In addition, this condition may increase the likelihood that the offspring is afflicted with a chronic disease [30]. It is now clear that maternal obesity is associated with many pregnancy complications, including gestational diabetes, preeclampsia and dystocia [31], and our previous study also documented differences in clinical characteristics of pregnant women between the overweight/obese group and normal-weight control group [8]. Moreover, maternal obesity can induce macrosomia in the newborns, and maternal pre-pregnancy and excessive gestational weight gain are always correlated with an increased risk of obesity as well as metabolic syndrome in the offspring during childhood [32]. Therefore, additional studies are warranted to identify how maternal obesity affects fetal and post fetal development and health.

Cord blood contains hematopoietic stem cells that can sustain the dynamic human hematopoietic and immune system function. These progenitor cell types are an essential source of hematopoietic stem cells for stem cell transplantation to treat several kinds of diseases. On the other hand, cord blood exchange between the fetal and maternal circulatory systems results in exchange of nutrients and metabolic end products. Accessing cord blood provides a means for monitoring the health of the fetus during its development in a clinical setting. One important evaluation involves karyotype analysis to check the fetal spine for abnormalities [33, 34]. Moreover, in recent years, it is now known that umbilical cord blood can diagnose the physical and mental health of the fetus. In one of these studies, it was reported that babies with an anomalous lipid level profile at birth may face a higher risk of social and psychological problems in childhood [35]. Therefore, cord blood testing can be employed to predict the psychological development of children. Another report showed that altered blood cytokine profiles in cord blood could be used as a potential biomarker to predict the risk of cerebral palsy in premature infants [36], and elevated IgE in cord blood may indicate an increased risk of disease in the future [37]. Thus, there is now convincing evidence suggesting that characterizing cord blood composition may be a predictor of postnatal health during development of the offspring.

Generally, implantation usually occurs in the uterus and the fetus obtains its nutrition and discharges its metabolic wastes via the umbilical vein and umbilical arteries, respectively. Undoubtedly, maternal health condition directly affects both the fetal and postnatal developments. Maternal obesity is one of important epigenetic changes. Such changes resulting in the alteration of gene expression patterns can disrupt both fetal phenotype as well as health at different developmental stages after birth. For instance, profile analysis of the maternal blood of obese individuals has identified high levels of lipid and leptin changes that are possible epigenetic modifiers of gene expression levels, which can alter the prenatal and postnatal fetal phenotype and health. An association was identified between increased leptin levels and adiposity at birth [38]. Another indication of epigenetic involvement being a determinant of phenotype and health during development stems from a comparison of the DNA methylation status in the fetal cord blood from obese pregnant women and normal weight subjects. The difference is thought to be a factor that accounts for the activation of the inflammatory signaling pathway in the obese subjects with an elevated BMI [39]. Such BMI rises were associated with hypomethylation of peripheral blood cells at genes involved in inflammatory and metabolic pathways in the offspring, which can last for several years [40]. Moreover, our results also showed that the outcome associated with this possible change in DNA methylation status is that the weight of the maternal obese offspring increased significantly more than that in the offspring of the age-matched normal weight control [8]. To confirm this association between DNA hypomethylation and altered metabolic status during and after pregnancy in the offspring of obese subjects, we determined if maternal obesity was associated with altered epigenetic control of the DNA methylation status in CpG islands of their fetuses and offspring. Such an assessment allowed us to next interrogate the underlying mechanism whereby DNA hypomethylation induces responses that account for phenotypic and altered health conditions in the offspring. The results showed that average methylation levels of cytosine in the CGI and promoter were significantly lower in the obesity group and the total DMRs were over 1000 between the two groups.

There are numerous DMRs on the X chromosome. During early human embryogenesis, a randomly selected X chromosome is epigenetically silenced in each female cell [41, 42]. In the current research, methylated cytosine analysis in DMR was performed on 14 cord blood samples. The samples were separated into a control group (3 male and 3 female newborns) and an overweight/obese group (4 male and 4 female newborns). Thus, the number of DMRs in our data was quite huge because of X-chromosome inactivation in our research [43]. Except for the X chromosome, the number of DMRs on chromosome 1 was the most abundant. Chromosome 1 is the largest of the human chromosomes with as many as 3141 genes, and it contains about 8% of the DNA in human cells [44]. Therefore, there were more DMRs on chromosome 1 than on any of the chromosomes. On the other hand, 6.2% of the DMRs were at CpG sites on chromosome 19. A hallmark feature of chromosome 19 is its unusually high gene density, which is characterized by its high GC content, high replication rate and high rearrangement rate. Importantly, some genes related to glycolipid metabolism are located on chromosome 19 and account for why they are always considered to be relevant in several diseases in humans such as familial hypercholesterolemia and non-insulin-dependent diabetes if these genes are abnormally expressed [45]. Moreover, our previous study also demonstrated that maternal obesity can alter the C19MC microRNAs expression profile in fetal umbilical cord blood [8]. Besides, C19MC is the largest cluster, which up until now was found to be located on chromosome19q13.4 [46]. Accordingly, maternal obesity may have a crucial impact on the epigenetic inheritance of chromosome 19.

The changes that we measured in the of DNA methylation status in fetal cord blood in overweight/obese pregnant subjects are in agreement with a previous study [4]. Our enrichment analyses also identified numerous interrelated pathways that are related to metabolism and disease. Some DMR-related genes or promoters that enrichment analysis identified include AR, IRAK1 and ARNT. They are functionally related to controlling metabolism or disease. The androgen receptor (AR) is a member of the steroid hormone receptor family. Mutations and amplifications of AR genes have been reported in prostate cancer and breast cancer. There is a two-way interaction between AR and micro-RNA (miRNA) in prostate cancer; androgens can up-regulate or down-regulate selected miRNAs, and in turn AR itself is a miRNA target. AR positive primary breast cancer is characterized by increased AR expression and a hormone-driven transcription program. Therefore, the AR expression is routinely monitored to establish a preliminary diagnosis and drug target for therapeutic clinical management of prostate and breast cancers [47, 48]. Interleukin 1 receptor associated kinase 1 (IRAK1) is a serine/threonine protein kinase that initiates the innate immune response against foreign pathogens through the Toll-like receptor (TLR) and IL1 receptor (IL1R) signaling. IRAK1 has been shown to be abnormally expressed in a set of tumors leading to tumorigenesis and progression. IRAK1 enhances cancer stemness and paclitaxel resistance in cancer [49, 50]. The aryl hydrocarbon receptor nuclear translocator (ARNT) is a member of the basic helix-loop-helix (bHLH)/PAS family of hepatic transcription factors. It is also known as the hypoxia-inducible factor 1β (HIF1β), which can regulate both glucose homeostasis and lipid metabolism in mice. Decreased ARNT levels may alter gluconeogenesis, lipogenic gene expression, and serum ketone content in the liver [51, 52]. Taken together, these marked declines in the methylation status at a large number of gene loci reflects numerous potential targets warranting additional study to identify how to selectively reverse changes in gene expression levels that underlie the altered phenotypes and health of the offspring of obese subjects.

Furthermore, GO analysis showed that maternal obesity induced methylation changes that were mainly associated with metabolic process and cellular process, which are in accordance with previous study [35]. This agreement also confirms that newborns from overweight/obese pregnant women were heavier than those from the control group. KEGG pathway analysis showed that these changes in maternal overweight/obese subjects have an impact on diseases, such as cancer and cardiomyopathy. An interesting result of the KEGG pathway analysis was the significant enrichment of the cardiomyopathy, such as arrhythmogenic right ventricular cardiomyopathy (AC). Approximately, half of AC cases can be attributed to known genetic mutations and AC is generally considered a hereditary cardiomyopathy. Thus, epigenetic mechanisms are gaining more attention as potential regulators of the aforementioned molecular mechanisms in arrhythmogenesis [53]. On the other hand, there are many reports enumerating a relationship between maternal obesity and increases in cancer in the offspring. Obesity is an independent risk factor for malignant tumors such as colon cancer and liver cancer [54, 55]. When mothers are obese during pregnancy, their children have a higher risk of cancer in childhood [56, 57]. In previous reports, maternal obesity increases the probability of liver cancer in the offspring, and this risk can become progressively worse during subsequent generations [7]. Moreover, maternal obesity alters the intrauterine environment which affects the growth and development of the fetus. It can permanently change the structure, function and metabolism of the fetus, and subsequently increase the risk of cardiovascular disease in the offspring [58, 59]. Besides, other studies have shown that obesity in pregnant women is related to a series of adverse health conditions in their offspring, including lifelong obesity, metabolic disorders, insulin resistance, hypertension, diabetes, dyslipidemia, behavior problems and asthma [5, 60].

The decreased trend in the expression levels of HDACs, DNMT1 and DNMT3b is consistent with lowering of the cytosine methylation status in both the promoter and CGI in the obesity group. Previous research showed hypomethylation of DNA promoters can lead to tumors [61–63]. DNA hypomethylation induced oncogene overexpression is one of the main mechanisms of carcinogenesis. The initial observation of the role of hypomethylation in carcinogenesis was its association with c-Ha-ras and c-Ki-ras hypomethylation in primary human cancers [64]. Compared with matched normal tissues, colorectal cancers are more significant in hypomethylation and promoter-specific DNA methylation at the whole genome level [65]. Moreover, several studies reported that the DNA of breast cancer tissue was significantly hypomethylated, and extensive DNA hypomethylation correlates significantly with the degree of disease progression and the histological grade of malignant tumors [66]. Notably, the decreased expression of DNMTs has an important impact on gene recombination and chromosome separation, which plays an important role in tumorigenesis [67]. For instance, since low expression of DNMT1 can reduce 5mC to about 10% of the normal level, DNMT1–deficient mice are more prone to suffer aggressive lymphoma and chromosomal instability [68, 69]. On the other hand, it was reported that Dnmt3b is involved in maintaining cytosine methylation in cancer. Loss of the function of Dnmt3b can accelerate mouse lymphomagenesis via upregulating the tumor modifier Ment [70]. Accordingly, these findings are also definitely consistent with our GO and KEGG analyses.

It should be noted that there are still some drawbacks to our study. For instance, the PAXgene Blood DNA kit was employed to isolate the genomic DNA. However, there are several types of cells in a cord blood sample, such as nucleated red blood cells, granulocytes, monocytes, natural killer cells, B cells, CD4 + T cells, and CD8 + T cells [71]. Cellular heterogeneity is critical in epigenetic research [72, 73]. Single-cell sequence analysis or cell type decomposition will be better choices for our future research [74, 75]. Next, our sample size is not large enough to identify all of the genes that were hypomethylated in the newborn cord blood samples obtained from the maternal obese subjects. Thus, the results of the current study still need to be validated by enrolling a larger cohort in the future. On the other hand, the incidence of obesity in an Asian population is lower than in occidental cultures according to the current WHO criteria [60]. Therefore, the criterion for assigning individuals in the normal and obese groups may need to change since in the Asian culture the percentage of normal weight individuals in the general population is much higher than that in many Western countries.

## Conclusions

Umbilical cord blood connectivity between the fetal and maternal circulation enables the exchange of fetal and maternal circulatory constituents with one another. In order to determine if this connectivity accounts for the epigenetic changes that underlie the increases in maternal BMI and their detrimental effects on fetal or offspring development, we compared the DNA methylation status in newborn cord blood samples obtained from obese and normal weight subjects immediately after birth. The results showed that obese pregnant women have lower methylation levels of cytosine in CGI and promoters, and the expression levels of HDACs and DNMTs also exhibited downward trends when compared with those in matched normal weight controls. Moreover, GO and KEGG analyses demonstrated that DMR-related genes and promoters were enriched in the metabolic processes that may account for declines in the health of the fetuses and offspring belonging to the obese pregnant group. This association indicates that maternal overweight/obesity may increase the risk of metabolic disorders, cancer and cardiomyopathy in the offspring.

## Availability of supporting data

The datasets used and analyzed during the current study are available from the corresponding author on reasonable request.

## Supplementary Information

**Additional file 1: Table S1. **Sequences of primers used for RT-qPCR analysis

**Additional file 2: Table S2. **Proportions of total methylated cytosine in the mCpG, mCHG and mCHH

**Additional file 3: Table S3. **DMR numbers in chromosomes

**Additional file 4: Table S4. **DMR-related genes between the normal weight control group and overweight/obese group

**Additional file 5: Table S5. **DMR-related promoters between the normal weight control group and overweight/obese group

**Additional file 6: Table S6. **Expressions of HDACs and DNMTs analyzed by RT-qPCR

## Data Availability

The datasets used and analyzed during the current study are available from the corresponding author on reasonable request.

## Abbreviations

C: Cytosine
CpG: Cytosine phosphate-guanine
CGI: CpG island
DMRs: Differentially methylated regions
BMI: Body mass index
TBS: Targeted bisulfite sequencing
HDACs: Histone deacetylases
DNMTs: DNA methyltransferases
HCC: Hepatocellular carcinoma
BSMAP: Bisulfite sequence MAPping
GO: Gene ontology
KEGG: Kyoto encyclopedia of genes and genomes
miRNA: Micro-RNA
AR: Androgen receptor
IRAK1: : Interleukin 1 receptor associated kinase 1
AC: Arrhythmogenic right ventricular cardiomyopathy
ARNT: Aryl hydrocarbon receptor nuclear translocator
HIF1*β*: Hypoxia-inducible factor 1*β*
